# Vitamin D and cancer mortality in elderly women

**DOI:** 10.1186/s12885-015-1112-5

**Published:** 2015-03-08

**Authors:** Germaine Wong, Wai Hon Lim, Joshua Lewis, Jonathan C Craig, Robin Turner, Kathy Zhu, Ee Mun Lim, Richard Prince

**Affiliations:** 1 Centre for Kidney Research, Children’s Hospital at Westmead, Westmead, Australia; 2 School of Public Health, Sydney Medical School, The University of Sydney, Sydney, Australia; 3 Centre for Transplant and Renal Research, Westmead Hospital Westmead, Westmead, Australia; 4 University of Western Australia School of Medicine and Pharmacology, Sir Charles Gairdner Hospital Unit, Perth, Australia; 5 Department of Renal Medicine, Sir Charles Gairdner Hospital, Perth, Australia; 6 Department of Endocrinology and Diabetes, Sir Charles Gairdner Hospital, Perth, Australia; 7 PathWest, Sir Charles Gairdner Hospital, Perth, Australia

**Keywords:** Vitamin D, Cancer mortality, Survival analyses

## Abstract

**Background:**

There is increasing evidence that vitamin D deficiency is a risk factor for cancer, however it remains uncertain whether vitamin D deficiency also predisposes to death from cancer. The aim of the study was to determine the association between serum 25-hydroxy-vitamin D (25 (OH) D) concentrations and cancer-specific mortality in a community-based cohort of older post-menopausal women.

**Methods:**

Cox proportional regression analyses were conducted to examine the association between serum 25 (OH) D concentrations and the risk of overall and site-specific cancer mortality in a cohort of elderly women.

**Results:**

Over a median follow-up time of 10 years, a total of 84 cancer deaths were observed. Women with lower serum 25 (OH) D concentrations were at an increased risk of cancer death, but not for incident cancer. The excess risk for cancer death was observed with serum 25 (OH) D concentration less than 64 nmol/L (the median value) [adjusted HR: 1.61 (95% CI: 1.02 - 2.54, p = 0.04]. For every 30 nmol/L reduction in serum 25 (OH) D concentrations, there was a 30% increase in the overall risk of cancer death [adjusted HR: 1.33; 95% CI: 1.03 – 1.72, p = 0.02]. The excess risk appeared to be site-specific and greatest in those with haematological cancers [adjusted HR: 2.13: 95% CI: 1.0 – 4.55, p = 0.05].

**Conclusions:**

In elderly women, lower serum 25 (OH) D concentrations appear to be an independent risk factor for cancer-specific mortality, but not a risk factor for the development of cancer.

**Electronic supplementary material:**

The online version of this article (doi:10.1186/s12885-015-1112-5) contains supplementary material, which is available to authorized users.

## Background

Vitamin D deficiency is increasingly recognised as an important global public health problem. There is a growing body of epidemiological evidence demonstrating an inverse relationship between lower serum 25-hydroxy-vitamin D (25 (OH) D) concentrations and adverse health outcomes in the general population [1,2]. Among postmenopausal women with serum 25 (OH) D concentrations less than 40 nmol/L, there was at least a two-fold increased risk of all-cause and cardiovascular-related mortality compared to women with 25 (OH) D concentrations greater than 64 nmol/L [3,4]. Observational studies have shown that lower serum levels of 25 (OH) D concentrations may also be linked to several types of common cancers such as breast, prostate, colorectal and skin [3,5-9]. Two controlled trials have assessed the impact of vitamin D supplementation and the incidence of cancer with conflicting results. The largest trial involved over 36,000 women randomized to combination calcium and vitamin D supplementation and matching placebo and found no significant effects on the incidence of cancer in post-menopausal women after an average of 7 years of follow up [10]. The other randomized controlled trial found a significant reduction in overall cancer risk by 60% with calcium and vitamin D supplementation in 1,179 healthy post-menopausal women with high baseline serum 25 (OH) D concentrations over a follow-up period of 4 years [11].

Observational studies have also produced conflicting results regarding the risk of cancer death and reduced serum 25 (OH) D concentrations. Several population-based cohort studies have shown a linear association between lower serum 25 (OH) D concentrations and an increased risk of overall cancer mortality [12-15], whilst another study reported no increased risk of cancer death associated with vitamin D deficiency [16]. Recently, a meta-analysis of prospective cohort studies demonstrated a strong, inverse and independent relationship between serum 25 (OH) D concentrations and non-vascular mortality. After adjustment for measured confounders, cause-specific analyses showed that there was a 16% reduction in cancer mortality for every 2-fold increase in baseline serum 25 (OH) D concentrations [17]. However, a more recent systematic review of prospective epidemiological studies has failed to demonstrate a consistent association between 25 (OH) D concentration and cancer mortality in the general population [18]. Previous studies, however, have not examined the threshold of serum 25 (OH) D concentrations that is associated with an increased in site-specific cancer deaths in older women, nor taken into consideration the competing risk of death when examining cancer-specific mortality. In this study, we aimed to determine the association between serum 25 (OH) D concentrations and cancer-specific mortality in a community-based cohort of older women.

## Methods

### Study population

Data were obtained from a cohort of elderly women recruited to a randomised controlled trial of oral calcium supplements to prevent osteoporotic fractures, the Calcium Intake Fracture Outcome Study (CAIFOS) Australian Clinical Trials Registry Registration Number: ACTRN012607000055404). Human ethics approval for the use of linked data for the project was provided by the Western Australian Department of Health Human Research Ethics Committee (DOHWA HREC), project number #2009/24. Full details of the recruitment methods are reported elsewhere [19]. In short, a total of 1500 women were recruited in 1998 and randomised to receive either 1.2 g of elemental calcium in the form of calcium carbonate or a matching placebo. Participants were followed over 10 years.

Baseline data were collected on self-reported medical and treatment histories, and the associated major risk factors such as smoking status and history of alcohol intake. Systolic and diastolic blood pressures were recorded using a mercury column manometer with an adult cuff after the participants had been seated and resting for at least 5 minutes. Baseline weight (using digital scales with participants wearing light clothes and no shoes), height (assessed using a stadiometer) and blood pressure (an average of 3 measurements) were obtained at recruitment. Blood samples were also taken for creatinine, albumin, calcium, phosphate, total cholesterol and triglyceride. Prevalent chronic kidney disease was identified using hospital records between 1980 and 1998.

### Assessment of exposure

Serum 25 (OH) D2 and D3 concentrations were determined using a validated LC-MS/MS (Liquid Chromatography Tandem Mass Spectrometry) method at the RDDT Laboratories (Bundoora, VIC, Australia). Between-run coefficients of variation (CVs) were 10.1% at a 25 (OH) D2 mean concentration of 12 nmol/L and 11.3% at a 25 (OH) D3 mean concentration of 60 nmol/L. Our exposure factor, serum 25 (OH) D concentration, is the combined serum 25 (OH) D2 and D3 concentrations.

### Western Australia data linkage system

The Western Australia Data Linkage System (WADLS) is a comprehensive, population-based linkage system connecting 40 years of data from over thirty health related datasets for Western Australian residents coded using International Classification of Diseases (ICD) codes. The linkage system provides a complete validated record of every participant’s primary diagnosis hospitalizations and up to twenty-one additional diagnosis hospitalizations codes and cause of death from the coded records of the death certificates or from cancer registry.

### Record linkage

The Human Ethics Committee of the University of Western Australia approved the study and written informed consent was obtained prior to recruitment. The linkage process, using probabilistic linkage software, was carried out at Data Linkage Western Australia. Using key identifiers such as age, gender, addresses, post-codes from the CAIFOS study cohort, the WADLS then created a unique data linkage key to records of the same person within the WADLS database and the process would take multiple passes through datasets using different arrangements of the data items at each pass.

### Assessments of deaths

Cancer and non-cancer deaths were provided by the WADLS as documented on the hospital death certificates and previous medical history, and the coded discharge diagnosis data including all public and private inpatient hospitalizations and deaths within Western Australia using the ICD 9^th^ and 10^th^ and the revision codes for malignancy (ICD-10-AM codes C15-26, C30-39, C43-58, C64-75), atherosclerotic vascular disease: ischemic heart disease excluding arrhythmias (ICD-9-CM codes 410–414 and ICD-10-AM codes I20-I25); heart failure (ICD-9-CM code 428 and ICD-10-AM code I50); cerebrovascular disease excluding hemorrhage (ICD-9-CM codes 433–438 and ICD-10-AM codes I63-69, G45.9) and peripheral arterial disease (ICD-9-CM codes 440–444 and ICD-10-AM codes I70-74).

### Statistical analyses

Statistical analyses were performed using Stata 11 (Stata Crop, 4905 Lakeway Drive College Station, Texas 77845, USA) and SAS 9.3 (SAS Institute Inc, 100 SAS Campus Drive Cary, NC 27513–2414, USA). Baseline characteristics of women with the different baseline serum 25 (OH) D concentrations were compared using student’s *t* test for means and chi-square for proportions (Table 1). The follow-up periods in survival analyses were defined from the time of first inclusion into the trial (from 1998 through 2008) to the time of death from cancer. People alive or died from other causes were censored at the end of the follow-up period (31^st^ December 2008) or date of death.

**Table 1:** **Baseline characteristics of participants (n = 1188)**

	25-hydroxy-vitamin D concentration (nmol/L)		*P values for differences*
	Above median (≥64) (n = 597)	Below median (<64) (n = 591)	
**Patient characteristics**			
Age at entry (years, mean, sd)	75.1 (2.7)	75.2 (2.7)	0.54
Body mass index (kg/m^2^, mean, sd)	26.8 (4.5)	27.4 (4.9)	0.02
Systolic blood pressure (mmHg, mean, sd)	138.0 (18.1)	137.4 (17.9)	0.57
Diastolic blood pressure (mmHg, mean, sd)	73.4 (11.0)	72.3 (10.7)	0.07
Previous and current smokers (n, %)	219 (35.1)	231 (37.3)	0.49
Diabetes (n, %)	36 (6.0)	46 (7.8)	0.24
Chronic kidney disease (n, %)	43 (6.9)	41 (6.6)	0.83
Cardiovascular disease (n, %)	104 (16.7)	90 (14.5)	0.29
Daily alcohol use (grams, mean, sd)	6.1 (9.0)	6.4 (9.6)	0.62
**Medication use (n, %)**			
Angiotension-converting enzyme inhibitors or angiotensin II receptor blockers	91 (14.6)	83 (13.4)	0.54
Beta-blockers	81 (13.0)	68 (11.0)	0.27
Diuretics	79 (12.7)	96 (15.5)	0.16
**Treatment with calcium supplements (n, %)**	314 (50.4)	332 (53.6)	0.27
**Seasons at enrolment (n, %)**			
Summer	7 (1.1)	19 (3.1)	<0.001
Spring	227 (36.4)	311 (50.2)	
Autumn	192 (30.8)	213 (34.4)	
Winter	197 (31.6)	77 (12.4)	
**Laboratory measurements**			
Creatinine (umol/L, mean, sd)	79.8 (15.9)	76.1 (14.9)	<0.001
Albumin (g/L, mean, sd)	44.0 (2.7)	43.6 (3.2)	0.004
Calcium (mmol/L, mean, sd)	2.35 (0.1)	2.32 (0.1)	0.005
Phosphate (mmol/L, mean, sd)	1.18 (0.1)	1.17 (0.1)	0.61
Estradiol	26.7 (14.6)	27.9 (17.8)	0.27
Estimated GFR (ml/min/1.73 m^2^, mean, sd)	64.9 (13.3)	68.3 (13.2)	<0.001

### Cause-specific analyses for cancer death

The proportion of participants alive was calculated using the Kaplan-Meier method. Univariate Cox regression models were developed to assess the risk factors of cancer mortality within the cohort. All explanatory variables that had an association with cancer death at P < 0.25 in the unadjusted analyses were included in the multivariable-adjusted analyses. Using a step-wise backward elimination process, the least significant variables were then removed from the base model. Only variables with P < 0.05 remained in the final parsimonious model. In all models, we adjusted for age, season, and smoking status. Potential effect modification was also tested between the study factor (serum 25 (OH) D concentrations) and all other covariates using the two-way interaction term. There were no effect modifications between serum 25 (OH) D concentration and other study factors. The proportional hazard assumptions of all Cox regression models were tested statistically and graphically by plotting the Schoenfield residuals. Site specific analyses were also performed to assess the relationship between reduced serum 25 (OH) D concentrations and the risk of the six most common cancer deaths within the cohort of elderly women. The serum 25 (OH) D concentrations were modelled as a continuous and categorical variable (less than and greater or equal than the median serum 25 (OH) D concentrations [<64 nmol/L or ≥ 64 nmol/L]). We also used fractional polynomials in the Cox regression model to evaluate the functional form of the association between the continuous variable of serum 25 (OH) D concentrations and overall cancer mortality. The hazard ratio for the difference between the serum 25 (OH) D concentrations and the median value of 64 nmol/L was estimated from the model coefficient and plotted to show the change in the risk of cancer death with lower and higher serum 25 (OH) D concentrations from the median value.

### Cause-specific analyses for cancer incidence

To determine whether the higher risk of cancer mortality was a reflection of the underlying cancer risk among those with reduced serum 25 (OH) D concentrations, we also assessed the association between overall cancer incidence and serum 25 (OH) D concentrations in the adjusted Cox regression model.

### Competing risk analyses

As a secondary analysis, we conducted a nonparametric estimation of the cumulative incidence of cancer mortality in participants with varying baseline serum 25 (OH) D concentrations, taking into account the informative nature of censoring due to competing risk. The cumulative incidence of cancer death is estimated using two main steps. We first considered the event of interests (i.e. cancer death) and other competing events such as vascular death as ‘events” and then calculated the Kaplan-Meier estimate of the overall “events”. Anyone who was not experiencing the “event” (i.e. event free) was considered censored [20].

### Sensitivity analyses

To ensure that all cancer diagnoses and subsequent deaths occurred before measurements of serum 25 (OH) D concentrations were taken, we had excluded participants who died from cancer within the first two years since the inception of the study in our sensitivity analyses. We also assessed the risk of cancer mortality among those with lower serum concentrations of 25 (OH) D (less than 46 nmol/L) compared to those with the highest quartile of serum 25 (OH) D concentrations (≥83 nmol/L).

## Results

From a total of 1500 participants, 228 (15.2%) with incomplete serum 25 (OH) D concentration and a further 84 participants (5.6%) with cancers diagnosed prior to inception of the study were excluded, leaving a total of 1188 participants in the final analyses. The median age, body mass index (BMI), and baseline serum 25 (OH) D concentrations of those included in the study were 75.1 (Interquartile range (IQR): 73 to 77) years, 26.5 (IQR: 23.8 to 29.7) kg/m^2^ and 64 (IQR: 46.2 to 83.2) nmol/L, respectively. The median follow-up period was 10 years (interquartile range: 0.41 years), resulting in 12,647 persons-years of follow-up.

### Baseline characteristics of the study participants

The baseline characteristics are shown in Table 1. A total of 591 (49.7%) participants had serum 25 (OH) D concentrations less than 64 nmol/L. Compared to those with serum 25 (OH) D concentrations above or equal to the median (≥64 nmol/L), participants with serum 25 (OH) D concentrations less than 64 nmol/L had lower BMI (p = 0.02), lower serum albumin (p = 0.004), calcium (p = 0.005), and a higher estimated glomerular filtration rate (p = 0.001) at baseline.

### Frequency and incidence of cancer death

A total of 274 deaths were observed during the follow-up period, with 84 (30.7%) and 124 (45.3%) cancer and vascular deaths, respectively. Cancers of the digestive system (27, 32.1) were the most common cause of cancer death, followed by lung (13, 15.4%), haematological (11, 13.0%), breast (6, 7.1%), and cancers of the central nervous system (5, 6.0%).

### Association between serum 25 (OH) D concentrations and cancer-mortality

**Figure 1:**
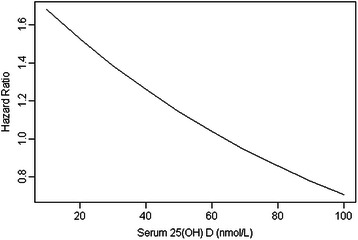
**Adjusted HR across continuous measures of serum 25 (OH) D concentrations.**

Table 2 shows the unadjusted and adjusted risk factors for cancer mortality. A significant increase in cancer mortality was observed in those with reduced levels of serum 25 (OH) D concentrations. Figure 1 shows how the adjusted hazard ratio (HR) for incident cancer death changes as the continuous measures of serum 25 (OH) D concentrations move further away from the median value of 64 nmol/L (which has an HR of 1). For every 30 nmol/L reduction in serum 25 (OH) D concentrations, there was a significant increase in cancer-specific mortality by 33% from a threshold of 64 nmol/L, after adjusting for the effects of age, seasonal changes, chronic kidney disease and smoking status (adjusted HR: 1.33; 95% confidence interval (CI): 1.03 – 1.72, p = 0.02). Compared to participants with serum 25 (OH) D concentrations greater than or equal to 64 nmol/L, the adjusted HR for cancer death among those with serum 25 (OH) D concentrations less than 64 nmol/L was 1.61 (95% CI: 1.02 - 2.54, p = 0.04). We have also shown that the best-fit fractional polynomial was linear in the adjusted model (Additional file 1).

**Table 2:** **Risk factors for cancer mortality**

Variables	Unadjusted HR	95% CI	P-values	Adjusted HR	95% CI	p-values
Patient characteristics						
Age (per SD increase)	1.05	0.86 – 1.28	0.64	1.02	0.84 – 1.25	0.83
Diastolic blood pressure (per 10 mmHg increase)	1.10	0.90 – 1.34	0.35	-	-	-
Systolic blood pressures (per 10 mmHg increase)	0.97	0.87 – 1.09	0.60	-	-	-
Previous and current smoker	1.45	0.97 – 2.17	0.07	1.80	1.17 – 2.77	0.008
Body mass index (per SD increase)	0.99	0.81 – 1.21	0.93	-	-	-
Daily alcohol use (per gram increase)	1.09	0.85 – 1.41	0.49	-	-	-
Co-morbidities						
Diabetes	1.32	0.61 – 2.86	0.48	-	-	-
Chronic kidney disease	2.37	1.27 – 4.50	0.007	2.43	1.28 – 4.62	0.007
Cardiovascular disease	1.30	0.74 – 2.27	0.36	-	-	-
Season at recruitment						
Spring	Ref	-	0.39	Ref	-	-
Summer	2.07	0.63 – 6.78		1.02	0.24 – 4.34	0.97
Autumn	1.48	0.87 – 2.54		1.71	0.99 – 3.00	0.06
Winter	1.17	0.70 – 1.96		1.27	0.75 – 2.14	0.37
Treatment allocation						
Calcium supplements	1.11	0.76 – 1.63	0.59	1.09	0.73 – 1.62	0.67
Laboratory measurements						
25 (OH) Vit D (nmol/L)						
>83	Ref	-	0.06	Ref	-	0.04
>64 - 83	1.95	1.02 – 3.74		2.23	1.09 – 4.58	
>46 - 64	2.27	1.21 – 4.27		2.55	1.24 – 5.26	
0 - 46	2.12	1.11 – 4.05		2.63	1.26 – 5.45	
Albumin (per SD increase)	0.95	0.79 – 1.13	0.53	-	-	-
Calcium (per SD increase)	1.01	0.83 – 1.22	0.93	-	-	-
Phosphate (per SD increase)	0.70	0.14 – 3.46	0.66	-	-	-
Estimated GFR (per SD increase)	1.03	0.84 – 1.27	0.78	-	-	-

### Cumulative incidence probability after adjustment for competing risk of death

**Figure 2:**
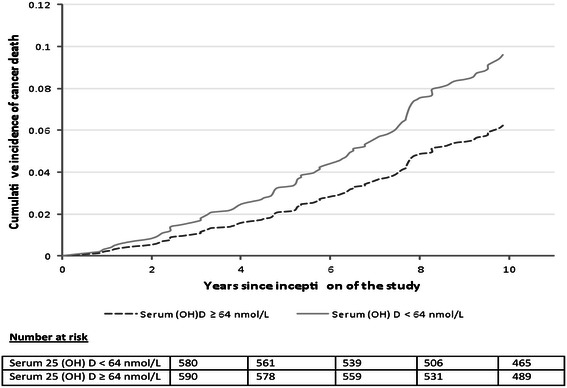
**Adjusted cumulative incidence of cancer mortality by serum 25 (OH) D concentrations.**

Figure 2 shows the adjusted cumulative incidence of cancer mortality and the serum 25 (OH) D concentrations (dichotomized below and/or above the group median value of 64 nmol/L) after adjusting for competing vascular deaths, age, smoking status, systolic and diastolic blood pressures. The cumulative incidence of cancer mortality among participants with 25 (OH) D concentrations < 64 nmol/L and ≥ 64 nmol/L 10 years after inception of the study were 0.096 and 0.062, respectively (p = 0.02).

### Site-specific risk of cancer death

**Figure 3:**
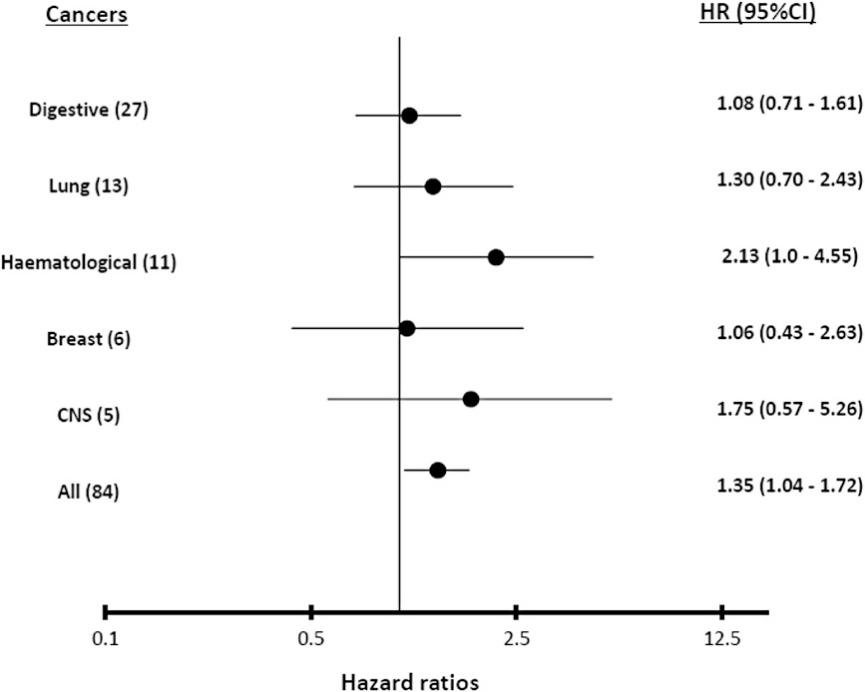
**Serum 25 (OH) D concentrations and site-specific cancer mortality.**

Figure 3 shows the selected site-specific HR for cancer death for every 30 nmol/L increase in serum 25 (OH) D concentrations. There was a significant association between reduced serum 25 (OH) D concentrations and increased risk of death from haematological cancers after adjusting for the effects of age, smoking status, seasonal changes and chronic kidney disease (adjusted HR: 2.13; 95% CI: 1.0 – 4.55, p = 0.05). This association was not significant in other cancer types including digestive, lung, breast, female genital and cancers of the central nervous systems.

### Sensitivity analyses

We also analyzed the cancer mortality risk comparing those with very low serum 25 (OH) D concentrations (less than 46 nmol/L) to those with baseline serum 25 (OH) D concentrations ≥ 83 nmol/L and found an increased risk of cancer death by at least 2.6-fold among those with very low serum 25 (OH) D concentrations (adjusted HR: 2.63; 95%CI: 1.26 – 5.45, p = 0.04). In addition, the risk of overall cancer death among those with serum 25 (OH) D concentration below the median (less than 64 nmol/L) remained significant after excluding participants who died from cancer within the first two years since inception of the study (adjusted HR: 1.59; 95% CI: 1.03 – 2.44, p = 0.04).

### Association between serum 25 (OH) D concentrations and cancer incidence

**Figure 4:**
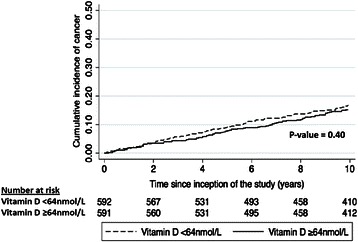
**Cumulative incidence of cancer by serum 25 (OH) D concentrations.**

A total of 191 incident cancers were observed during the follow-up period. The cumulative incidence of cancer among participants with serum 25 (OH) D concentrations < 64 nmol/L and ≥ 64 nmol/L ten years after inception of the study were 0.19 and 0.18, respectively (p = 0.60) (Figure 4). After adjusting for measured confounders, cancer risk was not significantly increased among those with serum 25 (OH) D concentrations below the median value (adjusted HR: 0.89, 95%CI: 0.67 – 1.18, p = 0.41, for those with 25 (OH) D levels less than 64 nmol/L).

## Discussion

In this large study of Australian women, with over 12,000 person-years of follow-up, we have shown that women with lower baseline were at an increased risk of overall cancer death but not for incident cancer. For every 30 nmol/L reduction in serum 25 (OH) D concentrations, there was an increased risk of cancer specific mortality by at least 30%, after adjusting for the effects of age, seasonal changes, smoking status and chronic kidney disease. The increased risk of cancer death also appeared to be site-specific, with the greatest risk of death from haematological malignancies observed among women with lower serum 25 (OH) D concentrations.

To our knowledge, this is a large population-based cohort study with the longest follow-up time to date investigating the association between serum 25 (OH) D concentrations and the overall and site-specific risk of cancer mortality in elderly women. Low serum 25 (OH) D concentrations have been shown to be associated with adverse clinical outcomes among those suffering from cancers. Previous ecological studies have reported breast cancer mortality was highest for cancer diagnosed in winter, the seasons with the highest serum 25 (OH) D concentrations [21]. Lower serum 25 (OH) D concentrations have also been associated with more advanced stages of breast cancers. More recently, a prospective cohort study of over 1300 older women suffering from breast cancer has shown that lower serum 25 (OH) D concentrations were linearly associated with 10% increased risk of cancer-related mortality and a 15% increased risk of disease recurrence for every 10 nmol/L decrement in serum 25 (OH) D concentrations [22].

Previous studies that have investigated the association between cancer mortality and serum 25 (OH) D concentrations have observed conflicting results. Several population-based cohort studies have reported an association between higher serum 25 (OH) D concentrations and reduction in overall cancer mortality [15,23,24]. In particular, significant inverse associations were observed for colorectal, pancreatic, oral and esophageal cancer mortality [12,25]. In contrast, results from the National Health and Nutrition Examination Survey (NHANES) study of over 16,000 participants, have shown no significant associations between serum 25 (OH) D concentrations and overall cancer mortality after adjustments for age, gender, smoking and ethnicity [16].

A significant association between haematological malignancies was detected among those with reduced serum 25 (OH) D concentrations. Although the exact biological rationale for the protective effects of vitamin D is unclear, findings from animal studies have shown that vitamin D can stimulate the production of a known antagonist of c-myc, a protein critical in promoting cell proliferation and the transformation of pre-malignant to malignant cells [26]. In addition, *in vitro* studies have also shown that the active metabolite of Vitamin D, 1,25 (OH) 2D, can inhibit the proliferation, angiogenesis and metastatic potential of tumour cells thereby suggesting a critical role of vitamin D in the control of cancer cell growth [27]. Studies have also reported a poorer prognosis among those with hypovitaminosis D in a cohort of patients with chronic lymphocytic leukaemia. The overall hazard for death is at least 1.6 times greater among patients with serum 25 (OH) D concentrations less than 25 ng/ml. *In vitro* studies have shown that treatment with Vitamin D analogues induces apoptosis of the mitochondrial pathway, therefore leading to inhibition of the proliferation of the B and T lymphocytes and lymphoma cell lines.

The higher serum 25 (OH) D concentration thresholds that predicted the increased risk of cancer mortality in our study cohort is unexpected. Most published literature described a cut-off point of 50 nmol/L and its association with the increased risk of overall and site-specific cancer death [28]. The higher than predicted threshold for the increased risk of cancer mortality may be due to chance or potential measurement errors such as regression dilution errors. Random measurement error in the exposure variable may potentially attenuate the association between vitamin D concentrations and cancer mortality at the pre-specified thresholds. This occurs because the measurements often fluctuate unpredictably around the true values, due to biological variations or imprecision of the measurement tool itself. The exposure factor, serum 25 (OH) D concentrations in our study, were measured only once at baseline, rather than repeatedly to minimize the potential errors. Our findings suggest future trials may consider higher doses of vitamin D to achieve a target serum 25 (OH) D concentration of 60 nmol/L or greater when evaluating the benefits and harms of vitamin D supplementation in older women with cancers.

Our study has a number of strengths. It is population-based, with very little missing baseline data, and over 12, 647 person years of follow up. Detailed data on confounders were also available. Using linked data from a mandatory state-wide cancer registry, we have accurately ascertained all cancer deaths since inception of the cohort.

Our study has a number of potential limitations. We excluded 17.1% of the original sample because of incomplete serum 25 (OH) vitamin D concentrations, but the number is relatively small, and those excluded have similar characteristics to those in the overall study population. The progressive change in serum 25 (OH) D concentrations over time and the risk of cancer death was not evaluated because only baseline serum 25 (OH) D concentrations were available. Findings from our study may not be generalisable to other populations such as the African Americans and Indigenous Australians because all participants are white post-menopausal Australian women. The lack of association between site-specific cancer mortality and serum 25 (OH) D concentrations may be due to the limited number of events for individual site-specific cancers, and therefore insufficient power to detect any significant differences in the risk of cancer deaths among women with lower and higher serum 25 (OH) D concentrations. Finally, although we had fully adjusted for all known risk factors for cancer death available in the dataset, residual confounding by other unmeasured socioeconomic and epidemiological factors such as physical activity and ultraviolet exposure may be present.

## Conclusions

In conclusion, lower baseline serum 25 (OH) D concentrations were independently associated with an increased risk of overall cancer mortality. However, the threshold of serum 25 (OH) D concentrations associated with the increased risk of cancer mortality may vary according to the characteristics of the study population. Additional prospective studies are required to investigate the association between serum 25 (OH) D concentrations and site-specific and overall incidence and mortality in older populations of both genders. Future carefully designed randomised controlled trials of vitamin D supplementation in older adults with cancers may be warranted to determine whether this association is causal and potentially reversible.

### Ethics approval

The Human Ethics Committee of the University of Western Australia approved the study and written informed consents were obtained from all participants. Approval number: 05/06/004/H50.

## Additional file

Additional file 1:
**Fractional polynomial model assessing the linearity assumption between serum 25 (OH) D concentrations and cancer mortality.**

## Abbreviations

25 (OH) D: 25-hydroxy-vitamin D
CAIFOS: Calcium Intake Fracture Outcome Study
CV: Coefficients of variation
WADLS: Western Australia Data Linkage System
ICD: International Classification of Diseases
IQR: Interquartile range
HR: Hazard ratio
NHANES: National Health and Nutrition Examination Survey
