# Supplementary material for: Vitamin D and cancer mortality in elderly women
Source: BMC Cancer. 2015 Mar 8;15:106. doi: 10.1186/s12885-015-1112-5 (PMC4356107; doi:10.1186/s12885-015-1112-5)
Supplement: Additional file 1: — Fractional polynomial model assessing the linearity assumption between serum 25 (OH) D concentrations and cancer mortality. [file 12885_2015_1112_MOESM1_ESM.doc]

**Additional file 1. Fractional polynomial model assessing the linearity assumption between serum 25 (OH) D concentrations and cancer mortality**

**
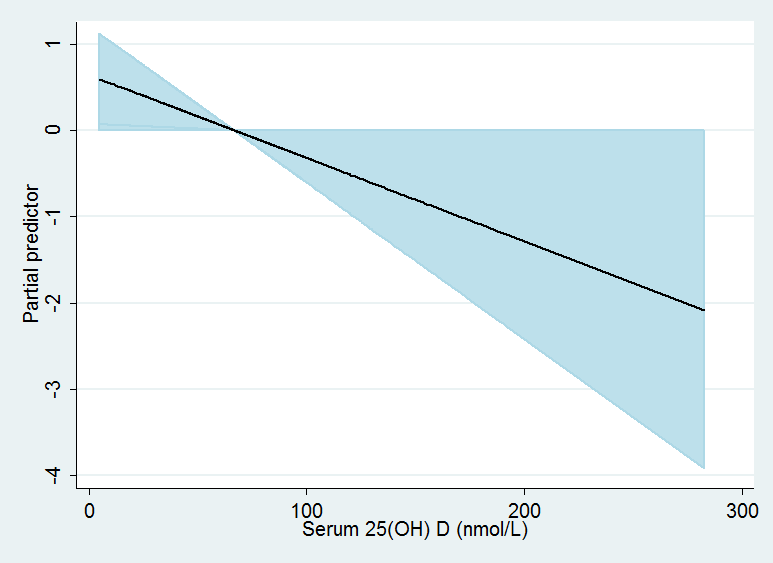
**
